# Identification of multivariable predictors for prediabetes: biochemical integration and pathophysiological mechanism analysis based on health examination data

**DOI:** 10.3389/fendo.2026.1895584

**Published:** 2026-08-05

**Authors:** Guitao Ruan, Xiaoliang Guo, Weizheng Zhang, Xiangsheng Cai

**Affiliations:** Clinical Laboratory, Guangzhou Cadre and Talent Health Management Center, Guangzhou, China

**Keywords:** apolipoprotein A1, inflammation, insulin resistance, pathophysiology, pre-diabetes, predictive model

## Abstract

**Background:**

Pre-diabetes is a critical, reversible intermediate metabolic state bridging normal glucose tolerance and overt type 2 diabetes mellitus (T2DM). Early clinical detection is essential for timely intervention, yet single-biomarker screenings often fail to capture the systemic metabolic and inflammatory alterations characteristic of this state. This study aimed to identify independent clinical and biochemical risk factors for pre-diabetes and to construct and validate a robust, low-cost multivariable predictive model leveraging routine health examination data, with a specific focus on dissecting the underlying molecular and biochemical pathways.

**Methods:**

A retrospective cross-sectional analysis was performed on clinical registry data from 2,376 adult participants (1,242 pre-diabetes patients and 1,134 normoglycemic controls) who underwent health examinations in 2023. Comprehensive demographic, anthropometric, and biochemical profiles were analyzed. Multivariable logistic regression was utilized to isolate independent risk and protective factors. Receiver Operating Characteristic (ROC) curve analysis evaluated the discriminative capacity of individual parameters versus the integrated multivariable model.

**Results:**

Univariate analyses demonstrated widespread systemic alterations across metabolic, inflammatory, hepatic, and lipid domains in pre-diabetic patients (P<0.05). Multivariable logistic regression identified gender(OR = 1.635, 95% CI: 1.177–2.274, P = 0.0034), age (OR = 1.105, 95% CI: 1.093–1.117, P<0.001), BMI (OR = 1.12, 95% CI: 1.079–1.163, P<0.001), MPV (OR = 1.103, 95% CI: 1.009–1.205, P = 0.0306), total protein (OR = 1.233, 95% CI: 1.076–1.416, P = 0.0029), γ-GT(OR = 1.007, 95% CI: 1.004–1.012, P<0.001), UA(OR = 1.002, 95% CI: 1.001–1.003, P = 0.0047), and apolipoprotein A1 (OR = 2.736, 95% CI: 1.200–6.273, P = 0.017) as independent risk factors. Serum creatinine, and high-density lipoprotein cholesterol (HDL-C) were identified as independent protective factors. While single parameters showed poor diagnostic capacity (AUCs <0.76), after bootstrap internal validation, the 8-parameter model achieved a bias-corrected AUC of 0.806, indicating good discriminative ability and robust generalizability.

**Conclusion:**

Pre-diabetes is associated with a complex, interconnected network of subclinical inflammation, hepatic stress, dyslipidemia, and altered skeletal muscle dynamics. Integrating these routine biomarkers into a unified predictive model offers a highly accessible, biologically sound, and cost-effective strategy for early diabetes screening in primary care settings.

## Introduction

The global prevalence of type 2 diabetes mellitus (T2DM) has reached epidemic proportions, presenting a major threat to public health and economic stability worldwide. According to the 10th Edition of the International Diabetes Federation (IDF) Diabetes Atlas, approximately 537 million adults aged 20–79 years are currently living with diabetes globally, a figure projected to rise to 783 million by 2045 (1). Crucially, this overt clinical cohort is preceded by an even larger, silently expanding population of individuals exhibiting pre-diabetes. Characterized by impaired fasting glucose (IFG) and/or impaired glucose tolerance (IGT), pre-diabetes represents an intermediate state of altered glucose homeostasis where glycemic parameters are elevated above physiological norms but remain below the diagnostic threshold for T2DM (2). Currently, more than 541 million adults worldwide are estimated to have IGT, with parallel projections estimating that more than 730 million individuals will be affected by 2045 (2). In China, rapid economic growth, dietary shifts toward hypercaloric foods, and increased sedentary behavior have driven the prevalence of pre-diabetes to between 35.7% and 50.1% among adults, representing a massive reservoir of potential T2DM cases (3–5).

At the molecular level, pre-diabetes is not a benign, static transitional phase, but rather an active pathophysiological state characterized by progressive tissue resistance to insulin coupled with a steady decline in pancreatic β-cell secretory capacity. Under physiological conditions, insulin binds to its transmembrane receptor, triggering the autophosphorylation of tyrosine residues and activating downstream cascades, most notably the phosphatidylinositol 3-kinase (PI3K)/Akt pathway. This cascade drives the translocation of glucose transporter 4 (GLUT4) storage vesicles to the plasma membrane in skeletal muscle and adipose tissues, facilitating glucose disposal (6).

In pre-diabetes, chronic nutrient overload and increased ectopic lipid storage disrupt this pathway. The accumulation of intracellular lipid metabolites, such as diacylglycerols (DAG) and ceramides, in non-adipose tissues (skeletal muscle and liver) activates novel protein kinases C (PKCs). These PKCs, alongside stress-activated kinases like c-Jun N-terminal kinase (JNK) and inhibitor of nuclear factor-κB kinase subunit β(IKKβ), promote the inhibitory serine phosphorylation (e.g., Ser307) of insulin receptor substrate 1 (IRS-1) (7). This phosphorylation blocks downstream PI3K/Akt activation, producing insulin resistance.

Initially, the pancreatic islets compensate through β-cell hyperplasia and hyperinsulinemia. However, chronic exposure to elevated glucose (glucotoxicity) and free fatty acids (lipotoxicity), combined with localized islet inflammation and endoplasmic reticulum (ER) stress, eventually triggers β-cell apoptosis and secretory exhaustion, leading to impaired glucose tolerance and overt hyperglycemia (8).

Beyond its role as a precursor to T2DM, pre-diabetes is independently linked to subvascular damage, endothelial dysfunction, accelerated atherogenesis, and increased risks of macrovascular and microvascular diseases. Chronic, low-grade systemic inflammation and oxidative stress—hallmarks of pre-diabetes—promote vascular cell adhesion molecule-1 (VCAM-1) and intercellular adhesion molecule-1 (ICAM-1) expression on endothelial cell surfaces. This facilitates monocyte infiltration, foam cell formation, and arterial wall remodeling before clinical hyperglycemia is diagnosed (9).

Fortunately, the progression from pre-diabetes to T2DM is not inevitable. High-quality clinical trials, such as the US Diabetes Prevention Program (DPP) and the Da Qing Diabetes Prevention Study, have demonstrated that intensive lifestyle modifications (dietary optimization and physical activity) or targeted pharmacotherapy (e.g., metformin) can reduce the risk of progression to T2DM by up to 58% (10). This highlights the critical importance of early identification and risk stratification.

The clinical challenge lies in selecting an effective screening strategy. The oral glucose tolerance test (OGTT) remains the gold standard for diagnosing IGT, yet its real-world utility is limited by high pre-analytical variability, the requirement for multi-hour clinical visits, and poor patient compliance. Fasting plasma glucose (FPG) is simple but fails to detect postprandial glycemic excursions, missing a significant portion of pre-diabetic patients. Glycated hemoglobin (HbA1c) is convenient and standardized, but its diagnostic accuracy is confounded by hemoglobin variants, nutritional anemias, alterations in erythrocyte lifespan, and higher cost, particularly in resource-limited clinical settings (11).

Routine annual health examinations offer an underutilized alternative. These screenings capture a broad array of physiological parameters—such as demographic data, anthropometric measurements, complete blood counts, hepatic and renal functional indices, and lipid profiles. While individual markers are often evaluated in isolation, they represent a highly integrated, multi-systemic response to early metabolic stress. Evaluating these routine parameters collectively using multivariable mathematical modeling could provide a low-cost, scalable, and clinically accessible method for early pre-diabetes screening.

The primary objective of this retrospective, cross-sectional study was to develop and validate a multivariable predictive model for pre-diabetes using routine clinical and biochemical data from a large health examination cohort in China. Additionally, we aimed to analyze the biological pathways and pathophysiological mechanisms linking these routine biomarkers to early glucose dysregulation.

## Materials and methods

### Study design and study population

This retrospective cross-sectional study used clinical and biochemical registry data from a health examination center in China. Data from 2,376 adult participants who underwent routine health checkups between January 1, 2023, and December 31, 2023, were evaluated.

- Pre-diabetes Group (n=1,242): Diagnosed based on the American Diabetes Association (ADA) criteria (12), which require a fasting plasma glucose (FPG) level between 5.6 mmol/L and 6.9 mmol/L (100–125 mg/dL) and/or a glycated hemoglobin (HbA1c) level of 5.7% to 6.4% (39–47 mmol/mol), in individuals with no history of glucose-lowering therapy. OGTT was not performed in the present study.
- Control Group (n=1,134): Normoglycemic individuals with FPG <5.6 mmol/L and HbA1c<5.7%, with no history of metabolic disorders or glucose intolerance.

Exclusion criteria were: a history of type 1 or type 2 diabetes mellitus; current use of insulin or oral anti-diabetic medications; severe renal impairment [estimated glomerular filtration rate (eGFR) <30 mL/min/1.73 m2]; liver failure or active cirrhosis; active malignancies; acute systemic infections or inflammatory states; pregnancy; or incomplete clinical or biochemical datasets. The study was conducted in accordance with the ethical guidelines of the Declaration of Helsinki.

### Clinical and laboratory measurements

All physiological measurements were taken by trained medical personnel using standardized protocols.

Anthropometric Metrics: Body weight and height were measured with participants barefoot and wearing light clothing. Body Mass Index (BMI) was calculated as weight in kilograms divided by height in meters squared (kg/m2). Waist circumference (WC) was measured at the midpoint between the lower rib margin and the iliac crest.

Hemodynamics: Systolic blood pressure (SBP) and diastolic blood pressure (DBP) were recorded using a validated oscillometric automated sphygmomanometer after participants had rested quietly in a seated position for at least 10 minutes.

Biochemical Analysis: Venous blood samples were collected via venipuncture in the morning following an overnight fast of ≥8hours. Complete blood counts—including white blood cell count (WBC), platelet count, and mean platelet volume (MPV)—were measured using an automated hematology analyzer (Mindray BC-6800Plus, Shenzhen, Guangdong, China).

Serum biochemical markers—including Fasting Blood Glucose(FBG), Aspartate Aminotransferase(AST), Alanine Aminotransferase(ALT), albumin (ALB), total protein (TP), γ-glutamyl transferase (γ-GT), serum creatinine (CR), uric acid (UA), total cholesterol (TC), triglycerides (TG), low-density lipoprotein cholesterol (LDL-C), high-density lipoprotein cholesterol (HDL-C), apolipoprotein B (ApoB), and apolipoprotein A1 (ApoA1)—were measured using a clinical chemistry analyzer (Canon Medical Systems Corporation TBA-FX8, Otawara, Tochigi, Japan) with manufacturer-specified reagents and calibrators.

Glycated hemoglobin (HbA1c) was measured using an automated HbA1c analyzer manufactured by TOSOH (Model HLC-723 G8).

### Statistical analysis

Continuous variables were tested for normality using the Kolmogorov-Smirnov test. Normally distributed variables are expressed as mean ± standard deviation (SD) and compared using the independent Student’s t-test. Non-normally distributed variables are presented as median and interquartile range (IQR) and compared using the Mann-Whitney U test. Categorical variables are expressed as frequencies and percentages (%) and compared using the Pearson chi-square (χ2) test.

To construct the predictive model, variables that showed statistical significance (*P* < 0.05) in univariate analyses, along with clinically relevant variables, were evaluated for multicollinearity using the Variance Inflation Factor (VIF). Variables with a VIF <5.0 were entered into a forward stepwise multivariable logistic regression analysis to identify independent risk and protective factors for pre-diabetes. The corresponding odds ratios (ORs) and 95% confidence intervals (CIs) were calculated.

Using the beta-coefficients derived from the final multivariable regression model, a predictive risk score formula was constructed for each participant. Receiver Operating Characteristic (ROC) curve analysis was then performed to evaluate and compare the diagnostic performance [Area Under the Curve (AUC), sensitivity, and specificity] of individual clinical parameters against the combined multivariable predictive model. Statistical significance was defined as a two-tailed *P* < 0.05. All statistical analyses were conducted using SPSS version 26.0 (IBM Corp., Armonk, NY, USA) and R software (version 2024.4.2.764).

To address potential overfitting and evaluate the internal validity of the final multivariable model, internal validation was conducted using the bootstrap resampling method with 1000 iterations. This procedure generated an optimism-adjusted Area Under the Curve (AUC) to estimate the model’s true discriminative performance. Additionally, model calibration was assessed to evaluate the agreement between the predicted probabilities and observed risks. This was achieved by visualizing a calibration plot and performing the Hosmer-Lemeshow goodness-of-fit test, where a *P*-value > 0.05 indicates adequate calibration.

## Results

### Baseline demographic and clinical characteristics

The study cohort consisted of 2,376 participants, divided into 1,242 pre-diabetes cases and 1,134 normoglycemic controls. The baseline demographic, physiological, and biochemical profiles of both groups are summarized in **Table 1** baseline_characteristics.

**Table 1:** Baseline characteristics of the participants.

Variable	Control	Prediabetes	P_value
Gender	399 (35.2)	313 (25.2)	<0.001
Age (years)	45.15 (11.89)	56.81 (12.09)	<0.001
Waist (cm)	81.06 (9.62)	87.05 (8.60)	<0.001
BMI (kg/m²)	23.94 (3.13)	25.69 (3.11)	<0.001
Systolic BP (mmHg)	120.55 (15.27)	130.89 (16.43)	<0.001
Diastolic BP (mmHg)	71.74 (10.61)	76.66 (11.03)	<0.001
RBC (×10¹²/L)	4.81 (0.50)	4.85 (0.49)	0.033
WBC (×10^9^/L)	5.94 (1.46)	6.46 (1.58)	<0.001
PLT (×10^9^/L)	247.47 (54.76)	242.80 (60.02)	0.048
HGB (g/L)	141.61 (15.14)	144.89 (13.09)	<0.001
MCV (fL)	90.22 (7.03)	91.26 (6.98)	<0.001
MPV (fL)	9.09 (1.21)	9.20 (1.14)	0.027
ALT (U/L)	23.93 (18.88)	27.26 (18.45)	<0.001
AST (U/L)	20.61 (7.44)	22.08 (8.56)	<0.001
TP (g/L)	73.88 (3.76)	74.49 (4.17)	<0.001
ALB (g/L)	45.26 (2.59)	45.21 (2.43)	0.589
GLB (g/L)	28.62 (3.34)	29.29 (3.84)	<0.001
GGT (U/L)	26.84 (23.43)	39.03 (43.75)	<0.001
Creatinine (μmol/L)	80.24 (25.35)	82.06 (19.16)	0.047
TG (mmol/L)	1.52 (1.36)	2.01 (1.81)	<0.001
LDL (mmol/L)	3.02 (0.84)	3.11 (0.97)	0.009
HDL (mmol/L)	1.47 (0.35)	1.37 (0.32)	<0.001
ApoA1 (g/L)	1.56 (0.28)	1.55 (0.27)	0.529
ApoB (g/L)	0.94 (0.24)	1.02 (0.27)	<0.001
UA (μmol/L)	392.64 (102.22)	423.88 (98.79)	<0.001

Univariate analyses revealed significant baseline differences between the groups. Participants in the pre-diabetes cohort were older, predominantly male, and had higher Waist, BMI, Systolic BP and Diastolic BP values compared to the controls (*P* < 0.001). Biochemically, the pre-diabetes group showed significantly elevated WBC count, HGB, MCV, MPV, ALT, AST, TP, GLB, serum albumin, γ-GT, TG, LDL, ApoB and uric acid (*P* < 0.001). Conversely, their HDL-C levels was significantly lower than those of the normoglycemic control group (*P* < 0.001).

### Multivariable logistic regression analysis

A multivariable logistic regression analysis was performed using forward stepwise selection to identify independent predictors of pre-diabetes while controlling for confounding variables (**Table 2**). No significant multicollinearity was observed among the variables (all VIFs <2.5).

**Table 2:** Multivariable logistic regression of risk factors for pre-diabetes.

Variable	OR	95% CI	P_value
Gender	1.635	1.177 - 2.274	0.0034
Age	1.105	1.093 - 1.117	<0.001
BMI	1.12	1.079 - 1.163	<0.001
MPV	1.103	1.009 - 1.205	0.0306
TP	1.233	1.076 - 1.416	0.0029
GGT	1.007	1.004 - 1.012	<0.001
Cr	0.979	0.971 - 0.986	<0.001
HDL	0.328	0.162 - 0.659	0.0018
ApoA1	2.736	1.2 - 6.273	0.017
UA	1.002	1.001 - 1.003	0.0047

The regression identified eight independent risk factors for pre-diabetes: Gender, Age, BMI, MPV, TP, γ-GT, uric acid, and ApoA1. Conversely, serum creatinine, and HDL-C were identified as independent protective factors. HDL-C and serum creatinine were significantly protective factors in preliminary screening but excluded from the final 8-parameter model due to non-retention in stepwise selection.

Notably, ApoA1 emerged as a strong independent risk factor in the regression model (OR = 2.736, 95% CI: 1.200–6.273, P = 0.017), contrasting with its absence of significant difference in unadjusted univariate analysis. This finding represents a statistical reversal paradox, which is explored in detail in the Discussion.

### Diagnostic performance of individual markers and the combined model

**Figure 1:**
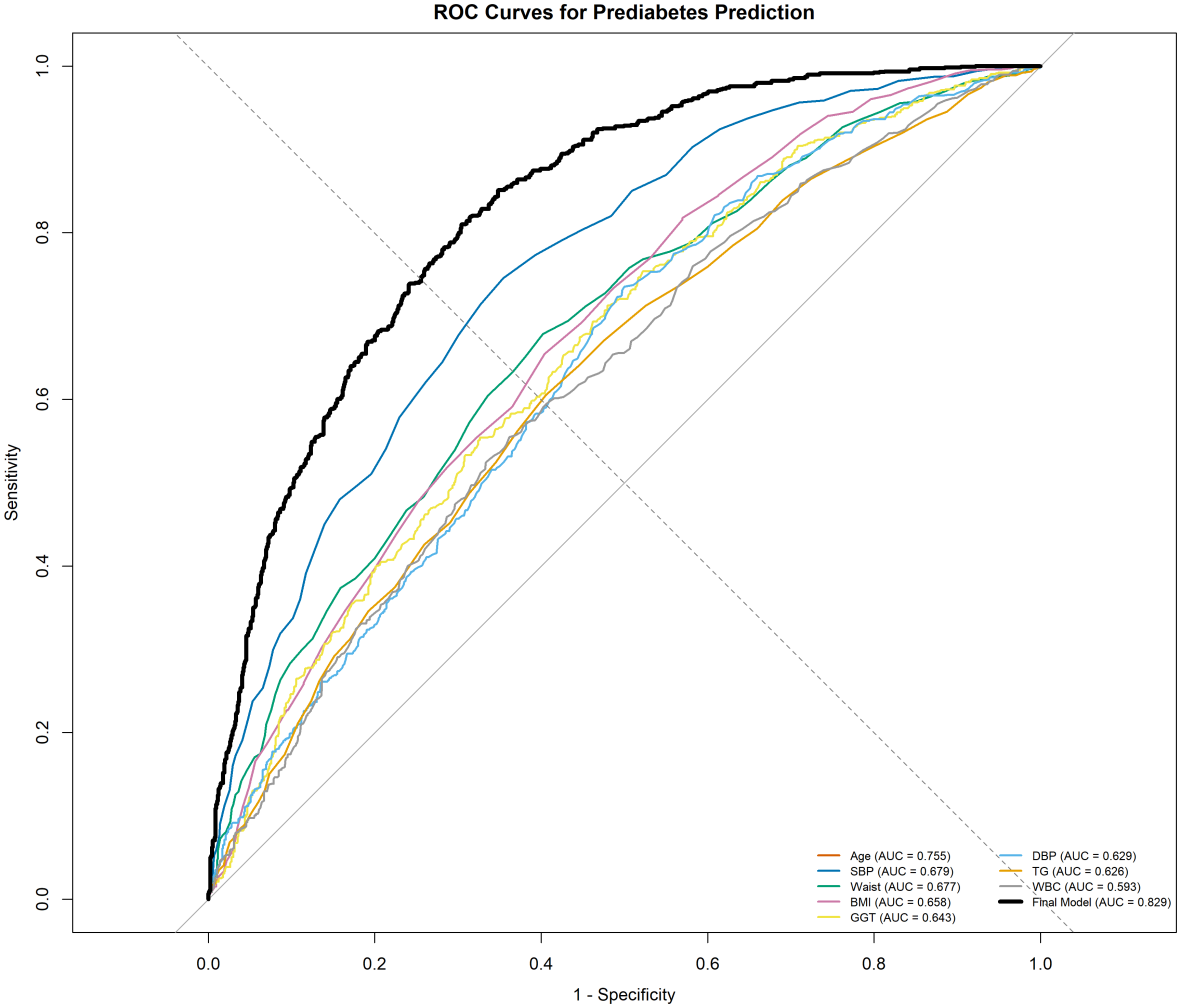
Receiver operating characteristic (ROC) curve of the prediction model for pre-diabetes.

To evaluate the discriminative power of these clinical metrics, ROC curve analyses were performed for individual variables and the combined 8-parameter logistic regression model (**Figure 1**).

Individually, none of the clinical or biochemical markers achieved an AUC >0.76, indicating poor single-parameter diagnostic performance. However, integrating the 8 independent demographic, physiological, and biochemical variables into a unified predictive model significantly improved performance, yielding an AUC of 0.829 (95% CI: 0.812–0.845), with a balanced sensitivity of 81.0% and specificity of 69.6% (**Table 3**).

**Table 3:** Diagnostic performance of selected individual markers and the combined model.

Predictive parameter/model	AUC	95% CI	Optimal cut-off	Sensitivity (%)	Specificity (%)
Age (years)	0.755	0.736 - 0.775	48.5	74.6	64.6
Systolic BP (mmHg)	0.679	0.658 - 0.701	123.5	67.9	59.8
Waist (cm)	0.677	0.656 - 0.699	83.5	65.5	59.6
BMI (kg/m²)	0.658	0.637 - 0.68	24	71.3	52
GGT (U/L)	0.643	0.621 - 0.665	19.95	73.5	50
Diastolic BP (mmHg)	0.629	0.607 - 0.651	73.5	60.5	59.4
TG (mmol/L)	0.626	0.603 - 0.648	1.445	55.5	63.8
WBC (×10^9^/L)	0.593	0.571 - 0.616	5.665	68.1	47.7
UA (μmol/L)	0.592	0.569 - 0.615	355.15	76.3	38.9
HDL (mmol/L)	0.584	0.561 - 0.607	1.385	61.4	54.4
Final Model(Apparent)	0.829	0.812 - 0.845	0.48	81	69.6
Final Model (Bootstrap validated)	0.806	0.805 - 0.808	0.505	75.0	71.7

### Internal validation and calibration of the combined model

To correct for potential overfitting and provide a more realistic estimate of model performance, internal validation was performed using 1000 bootstrap resamples. The optimism-adjusted AUC for the final 8-parameter model was 0.806.

**Figure 2:**
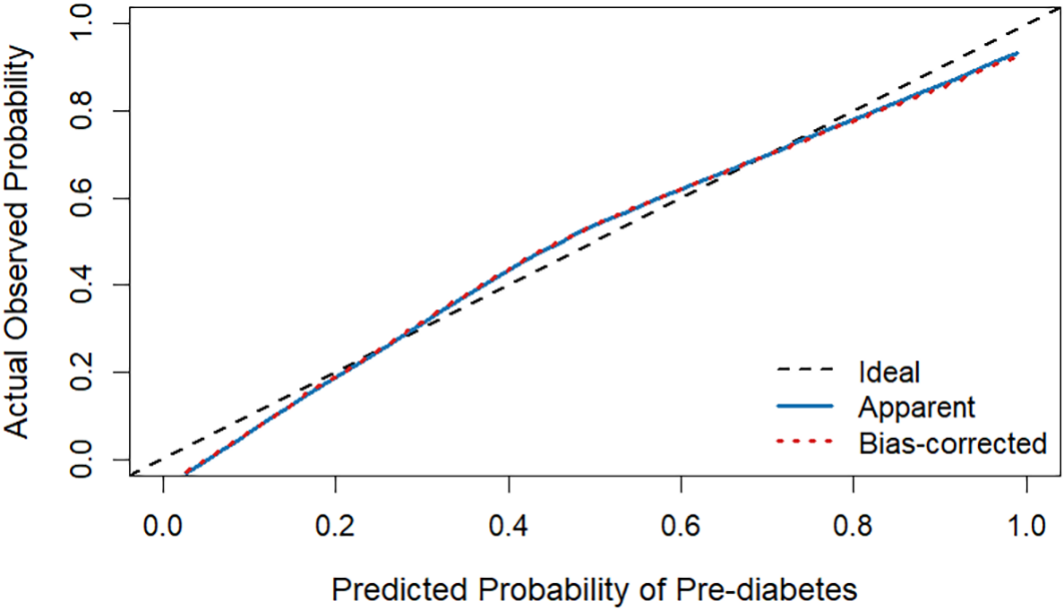
Calibration plot for the pre-diabetes risk model. The diagonal dashed line represents a perfect prediction. The red dotted line indicates the bootstrap bias-corrected calibration (B = 1,000), showing excellent agreement between predicted and observed probabilities.

The Hosmer-Lemeshow goodness-of-fit test yielded a *P*-value of <0.01 (χ^2^ = 31.396). While this suggests a statistical deviation, it is well-documented that the Hosmer-Lemeshow test is highly sensitive to large sample sizes, often resulting in significant P-values even when the clinical calibration is adequate. Therefore, we further evaluated the model’s calibration using a calibration plot with 1,000 bootstrap resamples (**Figure 2**). The calibration curve demonstrated visually excellent agreement between the predicted probabilities of pre-diabetes and the actual observed frequencies, indicating that the model is well-calibrated for practical application.

## Discussion

The primary clinical utility of this study lies in the development and validation of an 8-parameter predictive model for pre-diabetes using routine health examination data. This integrated model achieved an apparent AUC of 0.829, which remained robust at 0.806 after rigorous bootstrap internal validation, significantly outperforming individual physiological or biochemical markers (all individual AUCs < 0.76).

The baseline results demonstrate that pre-diabetes is not merely an asymptomatic increase in blood glucose, but rather a complex, systemic metabolic state. This state is characterized by low-grade chronic inflammation, lipid dysregulation, hepatic stress, altered skeletal muscle mass dynamics, and endothelial changes, all of which occur before the onset of overt diabetes.

### Demographic dimorphism and adipose-insulin interplay

Advanced age and elevated BMI were identified as strong, independent risk factors for pre-diabetes in this study, aligning with established metabolic epidemiological trends. Aging is closely linked to cellular senescence within pancreatic islets, progressive pancreatic β-cell apoptosis, and age-related sarcopenia, which reduces the primary physical sink for insulin-stimulated glucose disposal (13).

Our findings also revealed a pronounced sex-based difference, with males showing a 1.70-fold higher risk of pre-diabetes compared to females (OR = 1.635, P = 0.0034). This divergence is rooted in hormonal differences. Estrogens (particularly 17β-estradiol) bind to estrogen receptor alpha (ERα) on hepatocytes, skeletal myocytes, and adipocytes. This signaling enhances insulin receptor substrate-1 (IRS-1) activity and promotes the translocation of glucose transporter 4 (GLUT4) to the cell membrane (14). Estrogen also suppresses skeletal muscle inflammation by inhibiting the NF-κB pathway.

In contrast, androgens in males tend to promote visceral rather than subcutaneous adipose tissue deposition. Visceral adipose tissue is highly lipolytic and resistant to insulin’s antilipolytic effects, releasing high volumes of free fatty acids (FFAs) directly into the portal vein. This delivers lipid metabolites straight to the liver, driving hepatic insulin resistance and increased glucose output (15).

The independent risk associated with BMI (OR = 1.12, P<0.001) further highlights the metabolic impact of adipose tissue expansion. Hypertrophied adipocytes undergo lipid spillover when their storage capacity is exceeded. This lipid spillover may reflect ectopic fat deposition in the liver, skeletal muscle, and pancreas in the form of diacylglycerols (DAGs) and ceramides (16).

Within skeletal myocytes, elevated intracellular DAG levels activate novel protein kinase C theta (PKCθ). Once activated, PKCθ phosphorylates IRS-1 on serine residues (such as Ser307), preventing its association with the p85 regulatory subunit of PI3K. This disrupts downstream Akt phosphorylation, blocking the translocation of GLUT4-containing storage vesicles to the sarcolemma and causing peripheral insulin resistance (17).

### Systemic inflammation and hematological dynamics

Our model identified elevated elevated Mean Platelet Volume (MPV) as an independent risk predictor (OR = 1.103, 95% CI: 1.009–1.205, *P* = 0.0306), demonstrating the role of chronic, low-grade systemic inflammation and early endothelial dysfunction in pre-diabetes.

The link between subclinical inflammation and insulin resistance is well established. Under metabolic overload, visceral adipocytes and tissue-resident macrophages release pro-inflammatory cytokines, including tumor necrosis factor-alpha (TNF-α), interleukin-6 (IL-6), and monocyte chemoattractant protein-1 (MCP-1). These circulating cytokines stimulate intracellular stress-activated kinases, specifically IKKβ and JNK, in insulin-target tissues.

JNK directly phosphorylates the inhibitory Ser307 residue of IRS-1, disrupting insulin signaling. Concurrently, activated IKKβ phosphorylates the inhibitor of NF-κB (IκB), releasing the transcription factor NF-κB to translocate to the nucleus. This promotes the transcription of pro-inflammatory genes, creating a chronic inflammatory feedback loop that drives insulin resistance (18).

The positive association with MPV suggests subclinical platelet activation. Platelets express functional insulin receptors that, under normal conditions, increase nitric oxide (NO) production, decrease calcium influx, and suppress aggregation.

In insulin-resistant states, platelets become resistant to these inhibitory effects, leading to increased calcium mobilization, structural rearrangement, and the release of dense granules. This produces larger, more reactive platelets (reflected by a higher MPV) that contribute to subclinical endothelial injury, microvascular damage, and accelerated atherogenesis before the clinical diagnosis of T2DM (19).

### Hepatic stress and the hepatometabolic axis

The liver is central to maintaining glucose homeostasis by balancing glycogenesis, glycogenolysis, and gluconeogenesis. In our model, γ-GT (OR = 1.007, *P* < 0.001) and total protein (OR = 1.233, *P* = 0.0029) were independently associated with pre-diabetes, highlighting the hepatometabolic axis.

γ-GT is a membrane-bound enzyme responsible for the extracellular degradation of glutathione (GSH), the primary cellular antioxidant. Elevated serum γ-GT serves as a sensitive biomarker for hepatic steatosis and localized oxidative stress.

Accumulated hepatic lipids (specifically DAGs) activate novel protein kinase C epsilon (PKCϵ) in hepatocytes. PKCϵ inhibits the insulin receptor kinase domain, preventing insulin-stimulated glycogen synthesis and uninhibited gluconeogenesis. This leads to increased hepatic glucose output under fasting conditions, manifesting as IFG (20).

The elevation in γ-GT is also a compensatory cellular response to oxidative stress, helping to transport extracellular amino acids into hepatocytes to replenish depleted intracellular GSH pools (21).

Elevated TP is independently positively correlated with prediabetes risk, with effects mainly driven by increased globulin driven by inflammation rather than albumin, reflecting a synergistic metabolic disorder between inflammation and glycation stress under hyperglycemic conditions:

Anti-inflammatory Compensation: Under hyperglycemic conditions, non-enzymatic glycation reactions of proteins are enhanced, and the large production of advanced glycation end products (AGEs) accelerates the clearance of damaged proteins, promoting compensatory acceleration of protein turnover rate. The liver may increase protein synthesis to counter elevated systemic oxidative stress and lipid peroxidation (22).

Hemoconcentration: Pre-diabetic individuals often present with subclinical arterial hypertension, altered vascular permeability, and autonomic dysregulation, which can cause mild hemoconcentration and elevate measured concentrations of extracellular proteins like total protein.

### The lipid paradox: HDL-C versus apolipoprotein A1

Our analysis revealed a significant lipid paradox: while HDL-C was protective against pre-diabetes (OR = 0.328, *P* = 0.0018), its primary protein component, Apolipoprotein A1 (ApoA1), was identified as a strong independent risk factor (OR = 2.736, *P* = 0.017). This finding warrants both statistical and biochemical discussion.

Statistical Explanation: The Suppression Effect

In this study, apolipoprotein A1 (ApoA1) showed no significant difference between the two groups at baseline (P = 0.529), but after multivariate logistic regression correcting for confounding factors, it was identified as an independent risk factor for prediabetes (OR = 2.736, 95% CI: 1.2-6.273, *P* = 0.017). However, HDL-C and ApoA1 are highly collinear variables.

When both are entered simultaneously into a multivariable logistic regression model, they can produce a “suppression effect” or “reversal paradox” (similar to Simpson’s Paradox) (23). In this scenario, the model uses the residual variance of ApoA1—the portion of its variance that is independent of HDL-C—to predict pre-diabetes.

This statistical phenomenon suggests that the predictive value of these lipids is maximized when they are evaluated together: once the highly protective effect of functional, lipid-rich HDL particles (represented by HDL-C) is accounted for, the remaining variance in ApoA1 is positively associated with pre-diabetes risk.

Biochemical Explanation: Dysfunctional ApoA1 and HDL Particles

Beyond statistical explanations, there is a clear biochemical rationale for why elevated free or non-HDL-associated ApoA1 could indicate increased metabolic risk.

Under conditions of chronic low-grade inflammation and oxidative stress, circulating HDL and ApoA1 undergo chemical modifications that impair their function, converting them into dysfunctional, pro-inflammatory molecules:

Myeloperoxidase (MPO)-Mediated Oxidation: Mechanism speculation based on literature reports, MPO, an enzyme released by activated neutrophils and macrophages in inflammatory states, co-localizes with ApoA1 in vascular walls. MPO catalyzes the generation of reactive chlorinating and nitrating species (such as hypochlorous acid), which target specific tyrosine and tryptophan residues on ApoA1 (most notably Tyr192 and Tyr166) (24). This oxidatively modified ApoA1 loses its affinity for the ATP-binding cassette transporter A1 (ABCA1), impairing its ability to accept cellular cholesterol. This halts reverse cholesterol transport (RCT) and prevents the formation of mature, lipid-rich, protective HDL particles (25).

Advanced Glycation End-products (AGEs): In pre-diabetes, subclinical hyperglycemia leads to the non-enzymatic glycation of lysine residues on ApoA1. Glycated ApoA1 has a reduced half-life, is rapidly cleared by the kidneys, and exhibits an impaired ability to activate lecithin-cholesterol acyltransferase (LCAT). This prevents the conversion of nascent discoidal HDL into spherical HDL, leaving a pool of modified, lipid-poor ApoA1 that cannot perform its protective metabolic functions (26).

Pro-inflammatory Shifts: Dysfunctional ApoA1 can associate with acute-phase proteins like Serum Amyloid A (SAA), displacing normal anti-inflammatory proteins. This modified ApoA1-SAA complex can activate Toll-like receptor 4 (TLR4) on endothelial cells and macrophages, promoting the secretion of pro-inflammatory cytokines rather than suppressing them (27).

Thus, while high circulating levels of mature, functional HDL-C remain protective, an elevated level of modified, dysfunctional ApoA1 that cannot associate properly with HDL particles may serve as a marker of advanced oxidative stress, lipid peroxidation, and systemic inflammation.

### Renal markers, skeletal muscle dynamics, and uric acid

In our model, serum creatinine emerged as an independent protective factor against pre-diabetes (OR = 0.979, P<0.001). In patients with normal renal function, serum creatinine serves as a reliable surrogate marker for total skeletal muscle mass, as it is a metabolic byproduct of creatine phosphate breakdown in myocytes (28).

Skeletal muscle is the primary tissue responsible for insulin-stimulated glucose disposal, accounting for up to 80% of glucose clearance during an insulin infusion (29). A lower creatinine level often indicates sarcopenia or reduced skeletal muscle volume.

In the presence of obesity (sarcopenic obesity), this reduction in muscle mass significantly shrinks the physiological sink available for glucose disposal, exacerbating insulin resistance and increasing the risk of pre-diabetes (30).

Additionally, serum uric acid (UA) was identified as an independent risk factor (OR = 1.002, P = 0.0047). Elevated uric acid is a byproduct of purine metabolism, catalyzed by the enzyme xanthine oxidase (XO).

XO activation is a major source of intracellular reactive oxygen species (ROS) in vascular endothelial cells. These purine-derived ROS directly inhibit endothelial nitric oxide synthase (eNOS) by oxidizing its essential cofactor, tetrahydrobiopterin (BH4), which reduces the production of nitric oxide (NO) (31).

A reduction in bioavailable NO impairs microvascular blood flow and limits insulin delivery to skeletal muscle capillaries, impairing glucose disposal and promoting systemic insulin resistance (32).

### Clinical implications and diagnostic utility

The main advantage of this multivariable model is its clinical and economic feasibility. While advanced biomarkers like adiponectin, high-sensitivity C-reactive protein (hs-CRP), tumor necrosis factor receptors, and microRNAs can improve the accuracy of diabetes prediction models, their high cost and lack of standardization prevent their use in routine screenings, especially in primary care or resource-limited settings.

In contrast, our model relies entirely on 8 parameters that are already collected during standard annual health checkups. Using these existing metrics, the combined model achieved an AUC of 0.829, which is highly comparable to more complex models that require specialized biomarker testing.

By integrating this multivariable formula into Electronic Health Record (EHR) systems, clinical laboratories can automatically calculate a patient’s pre-diabetes risk score from routine blood draws. This risk-stratification tool can identify high-risk individuals and prompt early, targeted lifestyle interventions, helping to prevent progression to T2DM on a population scale.

## Study limitations

Several limitations of this study should be noted:

Cross-Sectional Design: Due to the observational nature of the study, we cannot establish direct causal relationships between the identified clinical markers and the development of pre-diabetes.

Single-Center Cohort: The data were retrieved from a single health examination center, which may introduce selection bias. External validation in geographically and ethnically diverse cohorts is needed to confirm the model’s generalizability.

Lack of Dynamic Glycemic Indices: Direct measures of insulin resistance, such as homeostatic model assessment of insulin resistance (HOMA-IR) or fasting insulin levels, were not available in this health examination registry.

Potential impacts of omitting the OGTT: Isolated impaired glucose tolerance (IGT, defined as isolated postprandial hyperglycaemia) may go undetected. Isolated IGT primarily manifests peripheral insulin resistance and early defects in insulin secretion, and exhibits stronger correlations with oxidative stress and vascular damage. Accordingly, the present study provides a conservative estimation of “non-stimulated hyperglycaemia”. Caution should be exercised when extrapolating these conclusions to the full spectrum of ADA-classified prediabetes. Comparisons with landmark trials that include OGTT measurements [e.g., the Diabetes Prevention Program (DPP) and China Da Qing Diabetes Prevention Study (Da Qing Study)] are limited.

Unmeasured Lifestyle Confounders: Important lifestyle factors, such as physical activity levels, specific dietary patterns, alcohol consumption, and family history of diabetes, were not captured in the database. Incorporating these variables in future studies could improve the model’s predictive accuracy.

## Conclusion

This study shows that pre-diabetes is associated with systemic changes across metabolic, inflammatory, hepatic, lipid, and renal domains. We developed and validated a 8-parameter predictive model using routine health examination data, achieving an AUC of 0.829. This combined model significantly outperformed any single clinical or biochemical biomarker.

By utilizing widely available, low-cost laboratory parameters, this model provides a practical, biologically sound tool for early pre-diabetes screening and risk stratification, supporting the primary prevention of type 2 diabetes.

## Data Availability

The original contributions presented in the study are included in the article/supplementary material. Further inquiries can be directed to the corresponding authors.

## References

[1] International Diabetes Federation . IDF Diabetes Atlas, 10th Edition (2021). Brussels, Belgium: International Diabetes Federation. Available online at: https://diabetesatlas.org/ (Accessed May 5, 2026).

[2] Saeedi P Petersohn I Salpea P Malanda B Karuranga S Unwin N . Global and regional diabetes prevalence estimates for 2019 and projections for 2030 and 2045: Results from the International Diabetes Federation Diabetes Atlas, 9th edition. Diabetes Res Clin Pract. (2019) 157:107843. doi: 10.1016/j.diabres.2019.107843 31518657

[3] Xu Y Wang L He J Bi Y Li M Wang T . Prevalence and control of diabetes in Chinese adults. JAMA. (2013) 310:948–59. doi: 10.1001/jama.2013.168118 24002281

[4] Wang L Peng W Zhao Z Zhang M Shi Z Song Z . Prevalence and treatment of diabetes in China, 2013-2018. JAMA. (2021) 326:2493–504. doi: 10.1001/jama.2021.22208 34962526 PMC8715349

[5] McMillin GA Morad AW Boyd JM Johnson-Davis KL Metz TD Smid MC . Biological testing and interpretation of laboratory results associated with detecting newborns with substance exposure. Clin Chem. (2024) 70:934–47. doi: 10.1093/clinchem/hvae018 38549034

[6] Leto D Saltiel AR . Regulation of glucose transport by insulin. Nat Rev Mol Cell Biol. (2012) 13:383–96. doi: 10.1038/nrm3351 22617471

[7] Petersen MC Shulman GI . Mechanisms of insulin action and insulin resistance. Physiol Rev. (2018) 98:2133–223. doi: 10.1152/physrev.00063.2017 30067154 PMC6170977

[8] Halban PA Polonsky KS Bowden DW Hawkins MA Ling C Mather KJ . β‑cell failure in type 2 diabetes: postulated mechanisms and prospects for prevention and treatment. J Clin Endocrinol Metab. (2014) 99:1983–92. doi: 10.1210/jc.2014-1425 24712577 PMC5393482

[9] Tabák AG Herder C Rathmann W Brunner EJ Kivimäki M . Prediabetes: a high‑risk state for diabetes development. Lancet. (2012) 379:2279–90. doi: 10.1016/S0140-6736(12)60283-9 22683128 PMC3891203

[10] Knowler WC Barrett‑Connor E Fowler SE Hamman RF Lachin JM Walker EA . Reduction in the incidence of type 2 diabetes with lifestyle intervention or metformin. N Engl J Med. (2002) 346:393–403. doi: 10.1056/NEJMoa012512 11832527 PMC1370926

[11] Gallagher EJ Le Roith D Bloomgarden Z . Review of hemoglobin A1c in the management of diabetes. J Diabetes. (2009) 1:9–17. doi: 10.1111/j.1753-0407.2009.00009.x 20923515

[12] American Diabetes Association . Classification and diagnosis of diabetes: Standards of Care in Diabetes—2024. Diabetes Care. (2024) 47:S20–42. doi: 10.2337/dc24-S002 PMC10725812 38078589

[13] Chang AM Halter JB . Aging and insulin secretion. Am J Physiol Endocrinol Metab. (2003) 284:E7–E12. doi: 10.1152/ajpendo.00366.2002 12485807

[14] Mauvais-Jarvis F Clegg DJ Hevener AL . The role of estrogens in control of energy balance and glucose homeostasis. Endocr Rev. (2013) 34:309–38. doi: 10.1210/er.2012-1055 23460719 PMC3660717

[15] Geer EB Shen W . Gender differences in insulin resistance, body composition, and energy balance. Gend Med. (2009) 6:60–75. doi: 10.1016/j.genm.2009.02.002 19318219 PMC2908522

[16] Shulman GI . Ectopic fat in insulin resistance, dyslipidemia, and cardiometabolic disease. N Engl J Med. (2014) 371:1131–41. doi: 10.1056/nejmra1011035 25229917

[17] Samuel VT Shulman GI . The pathogenesis of insulin resistance: Integrating signaling pathways and cellular structures. Cell. (2012) 148:852–71. doi: 10.1172/jci77812 22385956 PMC3294420

[18] Shoelson SE Lee J Goldfine AB . Inflammation and insulin resistance. J Clin Invest. (2006) 116:1793–801. doi: 10.1172/jci29069c1 16823477 PMC1483173

[19] Santilli F Simeone P Liani R Davì G . Platelets and diabetes mellitus. Prostaglandins Other Lipid Mediat. (2015) 120:28–39. doi: 10.1016/j.prostaglandins.2015.05.002 25986598

[20] Perry RJ Samuel VT Petersen KF Shulman GI . The role of hepatic lipids in hepatic insulin resistance and type 2 diabetes. Nature. (2014) 510:84–91. doi: 10.1038/nature13478 24899308 PMC4489847

[21] Whitfield JB . Gamma glutamyltransferase. Crit Rev Clin Lab Sci. (2001) 38:263–355. doi: 10.1080/20014091084227 11563810

[22] Roche M Rondeau P Singh NR Tarnus E Bourdon E . The antioxidant properties of serum albumin. FEBS Lett. (2008) 582:1783–87. doi: 10.1016/j.febslet.2008.04.057 18474236

[23] Tu YK Gunnell D Gilthorpe MS . Simpson's Paradox, Lord's Paradox, and Suppression Effects are the same phenomenon—the reversal paradox. Emerg Themes Epidemiol. (2008) 5:2. doi: 10.1186/1742-7622-5-2 18211676 PMC2254615

[24] Zheng L Settle M Brubaker G Schmitt D Hazen SL Smith JD . Localization of nitration and chlorination sites on apolipoprotein A‑I catalyzed by myeloperoxidase in human atheroma and associated oxidative impairment in ABCA1‑dependent cholesterol efflux from macrophages. J Biol Chem. (2005) 280:38–47. doi: 10.1074/jbc.M407019200 15498770

[25] Shao B Oda MN Oram JF Heinecke JW . Myeloperoxidase: an inflammatory enzyme for generating dysfunctional high density lipoprotein. Curr Opin Cardiol. (2006) 21:322–28. doi: 10.1097/01.hco.0000231402.87232.aa 16755201

[26] Nobecourt E Davies MJ Brown BE Curtiss LK Bonnet DJ Charlton F . The impact of glycation on apolipoprotein A‑I structure and its ability to activate lecithin:cholesterol acyltransferase. Diabetologia. (2007) 50:643–53. doi: 10.1007/s00125-006-0574-z 17216278

[27] Rosenson RS Brewer HB Ansell BJ Barter P Chapman MJ Heinecke JW . Dysfunctional HDL and atherosclerotic cardiovascular disease. Nat Rev Cardiol. (2016) 13:48–60. doi: 10.1038/nrcardio.2015.124 26323267 PMC6245940

[28] Baxmann AC Ahmed MS Marques NC Menon VB Pereira AB Kirsztajn GM . Influence of muscle mass and physical activity on serum and urinary creatinine and serum cystatin C. Clin J Am Soc Nephrol. (2008) 3:348–54. doi: 10.2215/CJN.02870707 18235143 PMC2390952

[29] DeFronzo RA Tripathy D . Skeletal muscle insulin resistance is the primary defect in type 2 diabetes. Diabetes Care. (2009) 32:S157–63. doi: 10.2337/dc09-s302 19875544 PMC2811436

[30] Srikanthan P Karlamangla AS . Relative muscle mass is inversely associated with insulin resistance and prediabetes. J Clin Endocrinol Metab. (2011) 96:2898–903. doi: 10.1210/jc.2011-0435 21778224

[31] Kanellis J Kang DH . Uric acid as a mediator of endothelial dysfunction, inflammation, and vascular disease. Semin Nephrol. (2005) 25:39–42. doi: 10.1016/j.semnephrol.2004.09.007 15660333

[32] Lanaspa MA Sanchez-Lozada LG Choi YJ Cicerchi C Kanbay M Roncal-Jimenez CA . Uric acid induces hepatic steatosis by generating mitochondrial oxidative stress. J Biol Chem. (2012) 287:40732–44. doi: 10.1074/jbc.m112.399899 23035112 PMC3504786

